# SymGRASS: a database of sugarcane orthologous genes involved in arbuscular mycorrhiza and root nodule symbiosis

**DOI:** 10.1186/1471-2105-14-S1-S2

**Published:** 2013-01-14

**Authors:** Luis Carlos Belarmino, Roberta Lane de Oliveira Silva, Nina da Mota Soares Cavalcanti, Nicolas Krezdorn, Ederson Akio Kido, Ralf Horres, Peter Winter, Günter Kahl, Ana Maria Benko-Iseppon

**Affiliations:** 1 Universidade Federal de Pernambuco, Centro de Ciências Biológicas, Departamento de Genética, Recife, PE, Brazil; 2 Molecular BioSciences, Goethe University of Frankfurt, Frankfurt am Main, Germany; 3 GenXPro GmbH, Frankfurt am Main, Germany

## Abstract

**Background:**

The rationale for gathering information from plants procuring nitrogen through symbiotic interactions controlled by a common genetic program for a sustainable biofuel production is the high energy demanding application of synthetic nitrogen fertilizers. We curated sequence information publicly available for the biofuel plant sugarcane, performed an analysis of the common SYM pathway known to control symbiosis in other plants, and provide results, sequences and literature links as an online database.

**Methods:**

Sugarcane sequences and informations were downloaded from the nucEST database, cleaned and trimmed with seqclean, assembled with TGICL plus translating mapping method, and annotated. The annotation is based on BLAST searches against a local formatted plant Uniprot90 generated with CD-HIT for functional assignment, rpsBLAST to CDD database for conserved domain analysis, and BLAST search to sorghum's for Gene Ontology (GO) assignment. Gene expression was normalized according the Unigene standard, presented as ESTs/100 kb. Protein sequences known in the SYM pathway were used as queries to search the SymGRASS sequence database. Additionally, antimicrobial peptides described in the PhytAMP database served as queries to retrieve and generate expression profiles of these defense genes in the libraries compared to the libraries obtained under symbiotic interactions.

**Results:**

We describe the SymGRASS, a database of sugarcane orthologous genes involved in arbuscular mycorrhiza (AM) and root nodule (RN) symbiosis. The database aggregates knowledge about sequences, tissues, organ, developmental stages and experimental conditions, and provides annotation and level of gene expression for sugarcane transcripts and SYM orthologous genes in sugarcane through a web interface. Several candidate genes were found for all nodes in the pathway, and interestingly a set of symbiosis specific genes was found.

**Conclusions:**

The knowledge integrated in SymGRASS may guide studies on molecular, cellular and physiological mechanisms by which sugarcane controls the establishment and efficiency of endophytic associations. We believe that the candidate sequences for the SYM pathway together with the pool of exclusively expressed tentative consensus (TC) sequences are crucial for the design of molecular studies to unravel the mechanisms controlling the establishment of symbioses in sugarcane, ultimately serving as a basis for the improvement of grass crops.

## Background

Among the most ancient symbiotic associations of plants are the intracellular arbuscular mycorrhiza (AM) with fungi of the phylum Glomeromycota that emerged approximately 475 million years (Myr) ago. Approximately 400 Myr later, the nitrogen-fixing root nodule symbiosis (RNS) with rhizobacteria (rhizobia) evolved in association with a subset of the dicotyledonous angiosperms (mostly legumes). The understanding of mechanisms involved in the evolution of AM and RNS has in recent years faced dramatic advances through genetic analysis of the plant host. AM symbiosis requires several of the genes identified in RNS, which are therefore referred to as common symbiosis (SYM) genes. It is evident that the common SYM pathway evolved in the context of AM and became secondarily involved in RNS [1]. Besides of being capable of AM symbiosis, sugarcane (*Saccharum officinarum*) associates with rhizhospheric, associative and endophytic nitrogen fixing bacteria [2], that posses unique features yet to be characterized, but as occurred with RNS, it is possible that this system of beneficial plant-microbial association evolved already in the context of AM symbiosis.

Sugarcane is an important crop around the world for the production of sucrose. Sugarcane derivatives and byproducts aroused interest with focus on ethanol production, which may replace up to 10% of the world's gasoline consumption in this decade, leading to a reduction of 50 tons of carbon emission per year [3]. Sugarcane is considered the most suitable tropical crop for biofuel production, but surprisingly high N fertilizer applications in main producer countries raise doubt about the sustainability of production and are at odds with a carbon-based crop. Interestingly, the amounts of N fertilizer in Brazil's sugarcane plantation are very low, although neither the yields nor the soil N reserves appear to diminish [4]. This is believed to be a consequence of the association of sugarcane with soil-borne fungi and nitrogen-fixing bacteria, that both play a critical role in nutritional and plant growth processes [5,6]. Molecular studies on these associations in sugarcane are lagging far behind studies in other plants. Thus, SymGRASS database is considered a first step towards linking the already available information for other plants to sugarcane, and also represent a platform for the development and design of molecular experiments to study arbuscular mycorrhiza and nitrogen-fixing symbiosis in sugarcane.

## Methods

SymGRASS relational database was implemented in mySQL and PERL CGI scripting. The sugarcane sequences used in this work were downloaded from the NCBI's dbEST [7]. Prior to assembly, the sequences were trimmed for vector contamination, poly A/T, and sequences containing more than 30% of Ns, and cleaned using Seqclean [8]. The data was assembled using TGICL v2.1 [9]. A super assembly was generated from the first one using a scaffolding step with the translation mapping method and the proteome of *Sorghum bicolor *as reference to assemble contigs belonging to a same coding region [10]. Functional annotation was accomplished through a BLASTx run against the UniprotKB downloaded from the Uniprot protein database [11]. Gene Ontology terms were assigned to the TCs based on their similarity to UniprotKB protein accessed through the BLASTx and mapped to Gene Ontology association (GOA) [12]. Conserved domain annotation was performed with the aid of rpsBLAST run against a locally formatted CDD database [13].

EST information files for the *S. officinarum *complex were downloaded from the NCBI dbEST. A PERL script was designed to collect the experimental information about tissue, organ and developmental stage sampling contained therein. After assembling, the numbers of reads building a contig as well as their sample of origin were retrieved with the aid of a script written in PERL. The expression abundance was normalized, following the formula r.10e5/n, where r represents the number of reads, and n is the total number of read in the particular library, since this is the presentation standard by Unigene. After normalization, the TCs were ranked according the percentage of EST in the libraries. The differential expression of a TC is statistically given as R-value proposed by Stekel and colleagues [14].

The sequences from the SYM pathway used as query were obtained from the NCBI non redundant protein databank according to the related literature searched. Accession number and links to the literature are available at http://symgrass.dyndns.org. The sequences were compared to the SymGRASS sugarcane sequence database through a tBLASTn search with a cut-off e-value of 10e-5. The original annotation performed for the TCs were adopted for the SYM gene candidates.

For the search of antimicrobial peptide, the entire PhytAMP database was downloaded and used as query in a tBLASTn search against the SymGRASS sugarcane sequence database with default parameters [15]. Also the original annotation performed for the TCs were adopted for the antimicrobial peptide candidates. The electronic differential display was generated with the Bioconductor R statistical package [16]. All the procedures were conducted in a server with 48 cores and 128 GB random access memory.

## Results and discussion

### SymGRASS design

**Figure 1:**
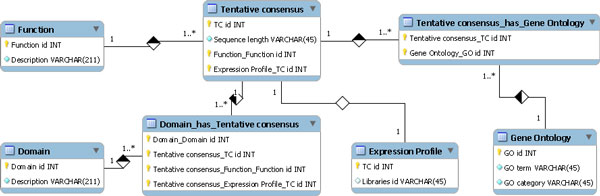
**Relational model of the SymGRASS database**.

**Figure 2:**
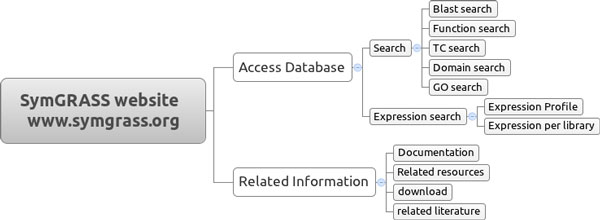
**SymGRASS web interface**.

SymGRASS is a relational database implemented in mySQL for organizing, storing and retrieving normalized information on symbiosis related genes in sugarcane and others grass crops (Figure 1). However the present release contains information just for sugarcane. We plan to add more organisms in the near future. Data were collected through public database search and curation as well as through literature review. The user can easily access and search the database through a web interface developed in PERL (Figure 2). The information on the database includes (I) sugarcane related sequence information: sugarcane Tentative Consensus (TCs) transcripts, expression profiles, putative functions, conserved domains and GO annotations; and (II) information about sugarcane orthologs of the genes in the common symbiosis pathway: gene name, accession, product, function, expression profile, conserved domains, GO annotation and link to related literature. The user can also query the database through BLAST service implemented with SequenceServer [17]. The available databases contain sugarcane TCs contigs, sugarcane TCs singletons and sugarcane-symbiosis-orthologs.

### Data

The sugarcane transcriptome has been interrogated under various conditions by several research groups around the world and sequenced using Sanger sequencing technologies, generating a significant amount of expressed sequence tags (ESTs) from various tissue samples. The resulting EST sequences are publicly available at the NCBI nucleotide databank - dbEST division [7]. SymGRASS Database provides sugarcane transcriptome sequence, annotation and expression data, along with special annotation for arbuscular mycorrhiza and root nodule symbiosis orthologs. The current release encompasses transcriptome sequences collected at the NCBI, available for various genotypes of the *S. officinarum *complex (Table 1). We will add more organisms and improve the assembly and annotation for the already provided data as soon as new studies and tools are published.

**Table 1:** EST libraries and sequence data available at SymGRASS.

Library Id	Tissue sample	Total Reads
SC34	Apical meristem and neighboring tissue (immature plants)	13,325
SC24	Apical meristem and neighboring tissue (mature plants)	10,813
SC19	Developing inflorescence 1 cm long	15,277
SC39	Developing inflorescence base 5 cm long	10,692
SC27	Developing inflorescence and rhachis 10 cm long	4,629
SC22	Developed inflorescence and rhachis 20 cm long	13,912
SC43	Developed inflorescence 20 cm long without rhachis	8,006
SC8	Developing seeds	17,012
SC31	Etiolated leaves from *in vitro *grown seedlings	4,543
SC37	First apical stalk internode of adult plants	6,906
SC5	First to third meristem internodes	1,078
SC25	Fourth apical stalk internode of adult plants	8,672
SC17	Sixth to eleventh internodes	7,234
SC35	Germinating sett roots	68
SC36	Lateral buds from field grown adult plants	5,904
SC6	Lateral buds from greenhouse grown adult plants	8,947
SC4	Leaf roll from field grown adult plants	15,141
SC3	Leaf roll including apex	3,581
SC9	Leaf whorl	4,316
SC1	Mature leaf tissue	6,867
SC23	Pool of calli exposed to low (4 °C) and high (37 °C) temperatures	8,978
SC10	Root	10,152
SC12	Root apex from adult plants	7,421
SC2	Root tips 0,3 cm long from adult plants	17,770
SC28	Shoot-root transition zone from young plants	7,839
SC18	Shoot-root transition zone from adult plants	12,806
SC45	Seedlings inoculated with *Glucoacetobacter diazotroficans*	14,629
SC11	Seedlings inoculated with *Herbaspirillum rubrisubalbicans*	9,684
SC21	Stem	11,379
SC41	Stalk bark from adult plants	13,151
SC16	Pool of tissues	2,490

Total		283,222

### *De novo* assembly

To obtain the best assembly with the publicly available sugarcane data, we used a combination of TGICL package with a subsequent scaffolding step using a translation mapping method and the proteome of *S. bicolor *as reference to assemble contigs belonging to a same coding region. This procedure generated 124,533 tentative consensus (TC) transcripts, designated as TC00001 to TC124533, the majority of which are singletons that may represent untranslated regions but could also contain reads originating from specific sugarcane genes for which no homologous sequences are present in sorghum. The putative function has been assigned to the transcripts based on their homology to known proteins in UniprotKB. GO terms have been assigned to the transcripts through mapping f the best BLASTx match in the UniprotKb to the Gene Ontology association files. This information can be accessed for each TC or a group of TCs on the SymGRASS online.

### Gene expression

EST information files for the *S. officinarum *complex were downloaded from the NCBI dbEST. The experimental information showed that several stages, tissues and organs from sugarcane were sampled and sequenced in the last decade. This collection enabled the delineation of an expression profile for sugarcane TCs, which is presented online as the percentage of a particular TC amongst all TCs. *R-value *indicates the significance of the observed differential expression be due to biological mechanisms. The user can access the expression profile for a TC over the libraries, for a group of TCs or for a particular library.

### Search of symbiosis related genes in the SymGRASS

**Figure 3:**
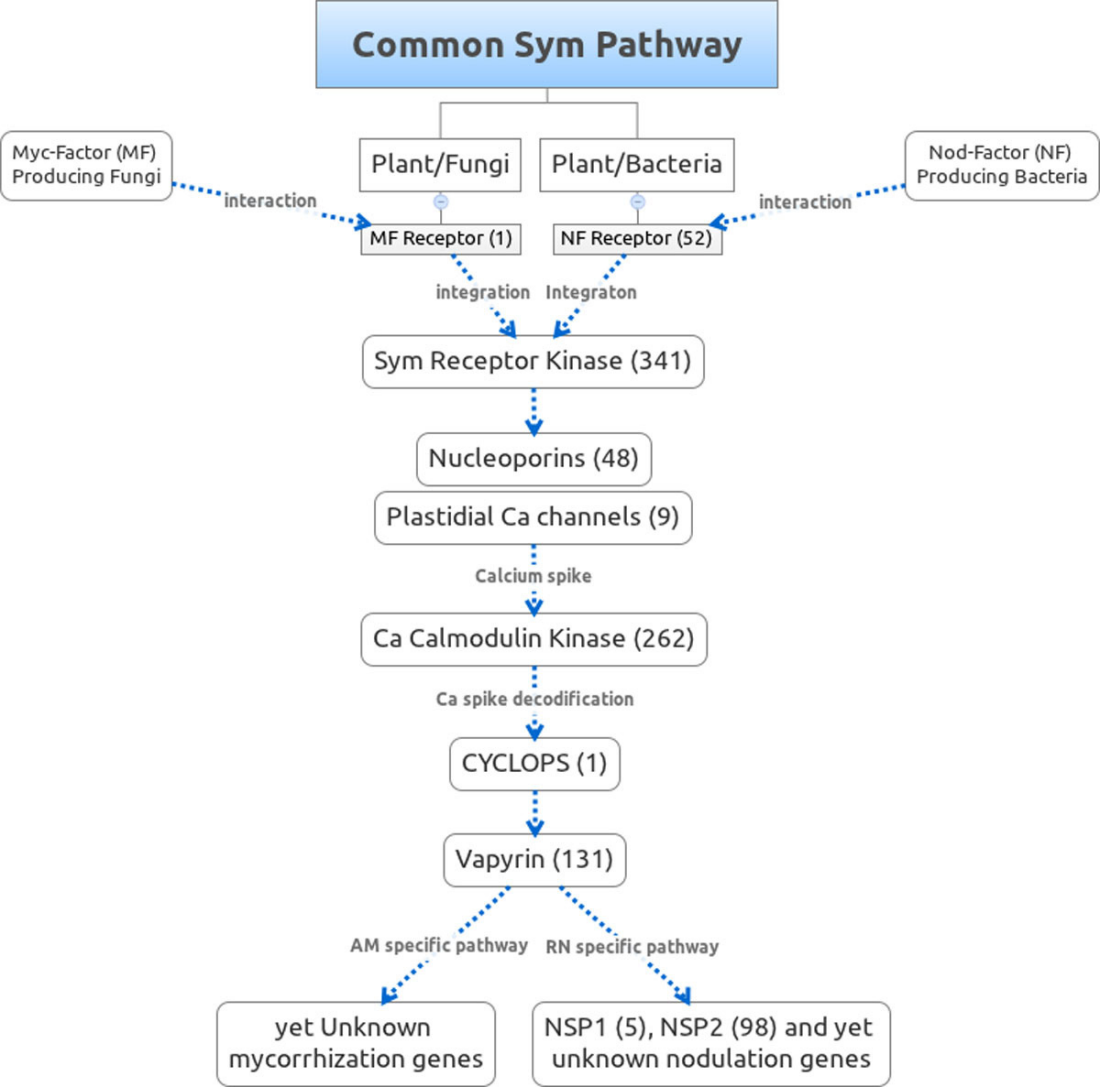
**Common SYM pathway for plant mycorrhization and nodulation with the numbers of sugarcane orthologs in parenthesis**. The representative protein in each node was collected from the literature. Accession numbers, journal links and detailed information for the query sequences and candidates are available online at http://symgrass.dyndns.org.

First we intended to access the extent of orthologs of the genes in the common SYM pathway required for AM and RN symbioses that are present in the sugarcane transcriptome. We defined the orthologous relationship on the basis of sequence similarity to legumes SYM genes and their monocot counterpart, if available, used as queries against the sugarcane *de novo *assembly through a tBLASTn search using an e-value as high as 10e-5. This search retrieved candidates TCs for all the nodes in the pathway (Figure 3). The detailed description of proteins accessions used as query for tBLASTn and related literature are available on-line along with their sugarcane SYM candidate sequences to download and also as a separate BLAST-searchable database at http://symgrass.dyndns.org.

**Figure 4:**
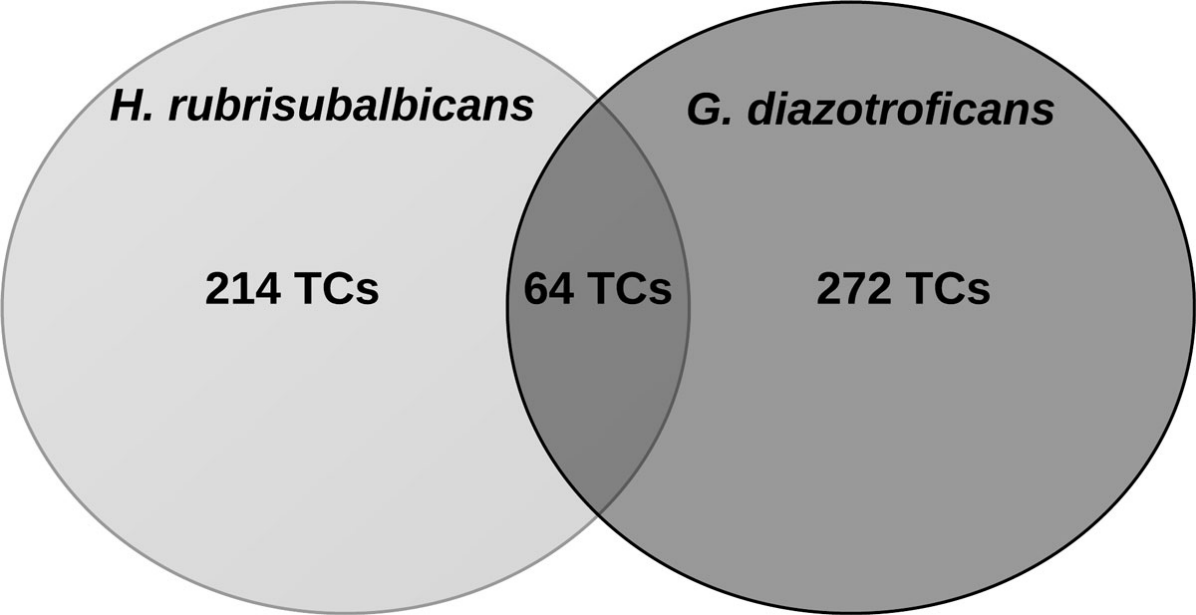
**Comparison of interaction sampling library transcripts with transcript profile of the other sampling libraries**.

We next compared the transcript expression profile of the library SC11 (seedlings interacting with *Herbaspirillum rubrisubalbicans*) and SC45 (seedlings interacting with *Gluconacetobacter diazotroficans*) with each other and with the transcript profiles of the other libraries. There were 550 genes exclusively expressed in these interactions with R-value greater than 8, 215 TCs appearing exclusively in the interaction with *H. rubrisubalbicans*, showing R-value between 15 and 90 (SC11), 272 TCs appearing just in the interaction with *G. diazotroficans*, presenting R-value between 8 and 43 (SC45), and 63 TCs common to both interactions, whose R-value was observed between 18 and 46 (Figure 4). In this regard one should mention that the two bacteria show different pattern of sugarcane entry and tissue colonization. While *H. rubrisubalbicans *colonizes mainly the leaves which are also the entry site, *G. diazotroficans *enters sugarcane through the roots and colonizes all tissues and organs, being already isolated from the endomycorrhiza network [5].

Out of the 214 exclusively expressed TCs in the SC11 library 134 were annotated to uncharacterized protein, 50 had no match to any protein at all and are considered sugarcane specific transcripts, and the remainder had annotation to defense genes, histones, phosphate transporters, actins and actin cap proteins, but the most interesting was the expression of a calcium calmodulin dependent kinase, a protein responsible for the Ca spike decodification in the SYM pathway [18], two transcription factors, and one Mutator-like transposon. Regarding the specific TCs observed in the library SC45, 171 out of 272 were annotated to uncharacterized proteins, 54 had no hit to known protein and rather represent sugarcane specific transcripts. It is also interesting to note that two other transcription factors and one retrotransposon were exclusively present in this library. Detailed annotation is available at http://symgrass.dyndns.org.

The TCs annotation for the exclusively expressed genes common to both interaction follows the same pattern as observed for the specific interactions with different symbionts. Of the TCs, 41 out of 64 TCs annotated to uncharacterized proteins, 8 TCs had no hit to known proteins, one TC was annotated as a transcription factor, and two TCs matched to retrotransposons proteins. To our knowledge, this is the first time that transposons and retrotransposons are found exclusively expressed in symbiotic interactions. How the transposable elements contribute to the establishment of the symbiotic interaction yet must be addressed.

**Figure 5:**
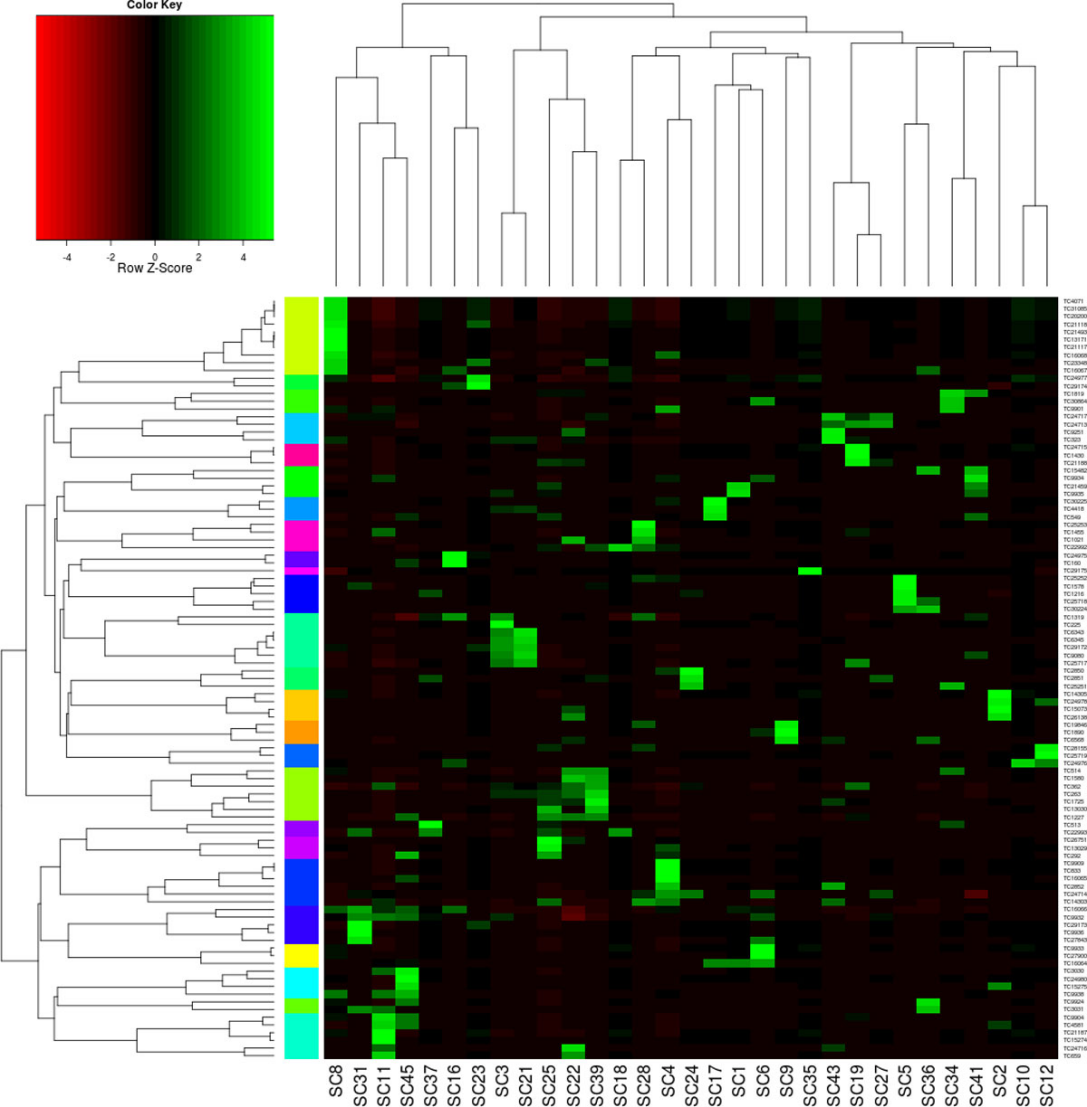
**Electronic differential display of AMPs in all libraries**. The leftmost colored column indicates cluster of genes with common expression profile. Library description and legend see Table 1.

Defense genes play a fundamental role in response to biotic interactions. It was thought that symbiotic interaction would rely on the down-regulation of defense response by the host plant [19]. But soon it became clear that the recognition of which microbial is friend or foe determines the outcome of a biotic interaction [20]. Antimicrobial peptides (AMPs) conceptually play an important role in the response to biotic interactions. Actually, more than 300 defensin-like proteins were predicted in nodules and seed from legumes species [21]. Thus, we attempt to access the information about this kind of molecules among sugarcane's transcribed genes. A tBLASTn search using the PhytAMP database as query against SymGRASS returned 99 AMP candidates. A differential display was generated for these AMPs candidates to compare their expression profile in the libraries. It became clear that rather than a down-regulation, a rearrangement is observed across the tissues sampled, especially regarding developing seeds and inoculated seedlings. 11 AMPs were strongly activated in the *H. rubrisubalbicans *inoculated seedlings in relation to the other libraries. 10 AMPs were activated in the interaction with *G. diazotroficans *and one nsLTP was exclusively expressed in symbiont challenged seedlings. It is also interesting to note that developing seeds were the most AMP rich tissue and that most of these AMPs were repressed in symbiont interacting seedlings (Figure 5).

### Present status and prospects

The present data curation status for the collected sequences in the SymGRASS database is summarized in Table 1. Links to research articles in the field of symbiosis are provided in the database concerning the common SYM pathway. We believe that the candidate sequences for the SYM pathway together with the pool of exclusively expressed TCs provide good background for the design of molecular studies to unravel the mechanisms controlling the establishment of symbioses in sugarcane. Since SymGRASS is an ongoing effort, we will continue to curate and consistently update the database as soon as new or updated data/tools are made available. We also plan to include other grass species such as *Sorghum bicolor, Zea mays, Oryza sativa, Setaria italica *and *Brachypodium distachyon*. Additional to more plants we intend to incorporate also bacteria and fungi in the database, and provide a routine to compare genes and grass/microorganism interactions between different species. As species are being added and the amount of publications concerning symbiosis increases, data curation will become more challenging. We expect that SymGRASS becomes a community-based database.

## Conclusions

SymGRASS regards the first web based data repository of molecular, cellular and physiological information on grasses and their symbionts, consisting of a user friendly resource available for plant biologists, microbiologists and breeders. The available annotated and assembled candidate sequences for the SYM pathway will help the recognition and manipulation of members involved in mechanisms by which sugarcane (and in the future other grasses) controls establishment and efficiency of endophytic associations.

## List of abbreviations used

AM: arbuscular mycorrhiza; GO: gene ontology; Myr: million years ago; RN: root nodule; RNS: root nodule symbiosis; SYM: common symbiosis pathway; TC: tentative consensus.

## Competing interests

The authors declare that they have no competing interests.

## Authors' contributions

LCB conceived of the study, and participated in its design and coordination, and draft the manuscript. LCB, RLOS and NMSC carried out data acquisition, categorization, organization and scripts for annotation. LCB and NK developed the web-site and tool integration. RH, EAK and PW helped in data curation and discussion. GK and AMBI helped in the definition of the experimental design and critical review. AMBI coordinated the research.

## Declarations

The publication costs for this article were funded by the corresponding author's institution.

This article has been published as part of *BMC Bioinformatics *Volume 14 Supplement 1, 2013: Computational Intelligence in Bioinformatics and Biostatistics: new trends from the CIBB conference series. The full contents of the supplement are available online at http://www.biomedcentral.com/bmcbioinformatics/supplements/14/S1.
